# FGAN@PB NP Nanozyme-Based Colorimetric–Photothermal Dual-Mode Immunosensor for Malachite Green Detection

**DOI:** 10.3390/bios15110719

**Published:** 2025-10-30

**Authors:** Min-Fu Wu, Jing-Min Li, Sha Li, Min-Hua Wu, Ri-Sheng Chen, Yan-Can Liu, Jian-Nan Liu, Zhen-Lin Xu, Yi-Chao Yang, Jia-Dong Li, Qing-Yi Lei, Si-Min Zhan, Lin Luo

**Affiliations:** 1 Department of Food Science, Foshan Polytechnic, Foshan 528137, China; wmf@fspt.edu.cn (M.-F.W.); liuyancan@fspt.edu.cn (Y.-C.L.); liujiannan@fspt.edu.cn (J.-N.L.); ljdong135@fspt.edu.cn (J.-D.L.); lqy2025@fspt.edu.cn (Q.-Y.L.); zsm2025@fspt.edu.cn (S.-M.Z.); 2 Guangdong Provincial Key Laboratory of Food Quality and Safety, College of Food Science, South China Agricultural University, Guangzhou 510642, China; lijm@bioeasy.com (J.-M.L.); 20241145003@stu.scau.edu.cn (R.-S.C.); xzlin@scau.edu.cn (Z.-L.X.); 3 School of Health Sciences Research, Research Institute for Health Sciences, Chiang Mai University, Chiang Mai 50200, Thailand; 4 Department of Histology and Embryology, Guangdong Medical University, Zhanjiang 524023, China; wugdmczp@gdmu.edu.cn; 5 School of Public Health, Guangzhou Medical University, Guangzhou 511436, China; yichaoyang@gzhmu.edu.cn

**Keywords:** immunosensor, nanozyme, malachite green, colorimetric–photothermal

## Abstract

In this study, a colorimetric–photothermal dual-mode immunosensor based on Fe(Ⅲ)–gallic acid composite Prussian blue nanozyme (FGAN@PB NPs) was developed for the highly sensitive detection of malachite green (MG) in aquatic products. This strategy addresses the stability limitations associated with conventional horseradish peroxidase (HRP). In the colorimetric mode, the immunosensor exhibited an IC_50_ of 7.56 ng/mL with a linear detection range of 2.21–25.84 ng/mL. In the photothermal mode, the linear range was 0.262–25.6 ng/mL, with a detection limit (LOD) of 0.31 ng/mL. The results from the two detection modes were mutually corroborative. Moreover, the detection of the proposed immunosensor was strongly correlated with the LC-MS/MS, offering a promising approach for the rapid on-site screening of MG and improving its applicability in complex sample matrices.

## 1. Introduction

Malachite green (MG) is a triphenylmethane-based industrial dye [1], which was illegally employed in aquaculture to prevent water mold infections and enhance the coloration of fish [2], owing to its strong antibacterial and antiparasitic properties [3]. However, MG and its primary metabolite, leucomalachite green, have been classified as Group 2B carcinogens by the International Agency for Research on Cancer (IARC) [4], with reported carcinogenic, teratogenic, and neurotoxic effects [5]. These compounds may bioaccumulate in the human body through the food chain, potentially causing irreversible health damage [6,7]. As a result, many countries have banned their use in food products [8]. In 2000, the European Union prohibited the application of MG in edible fish [9], and China has enforced stringent regulations banning the use of MG and crystal violet in aquaculture [10], requiring non-detectable levels in products [11]. Nevertheless, economic incentives continue to drive illegal usage, posing significant health risks to consumers [12].

Conventional detection methods such as high-performance liquid chromatography (HPLC) and liquid chromatography–mass spectrometry (LC-MS) offer high sensitivity but are hindered by expensive instrumentation [13,14,15,16], complex procedures, and extended analysis times [17], rendering them unsuitable for routine monitoring and rapid on-site screening [18]. The enzyme-linked immunosorbent assay (ELISA), characterized by high specificity and operational simplicity [19,20], is widely recognized as a benchmark technique in clinical diagnostics, and it also serves as an effective complementary tool for large-scale, on-site screening in food safety monitoring [21,22]. However, the traditional ELISA suffers from several limitations that hinder its broader application, including a dependence on natural enzymes, single-signal output, and vulnerability to interference from complex sample matrices [23].

Recent progress with nanozyme labels—especially Prussian blue (PB)-based peroxidase mimics—offers improved operational stability, a lower cost, and compatibility with colorimetric readouts [24]. Yet, many reported assays still operate in a single detection mode, which can limit result reliability in heterogeneous food matrices [25]. Moreover, ensuring efficient and stable antibody conjugation to catalytic labels remains essential to preserve activity and maximize assay performance [26].

In this work, we introduce an FGAN@PB NP label that integrates PB nanozyme domains with a gallic-acid-based scaffold to support robust antibody coupling and catalytic accessibility (without invoking additional mechanistic roles). Building on the strong NIR absorption of PB and the ox-TMB chromogenic reaction, we establish a colorimetric–photothermal dual-mode immunosensor for MG. The two readouts are mutually corroborative, mitigating single-mode bias, and the results show a strong correlation with LC–MS/MS in fish and aquaculture-water samples, underscoring the method’s suitability for rapid on-site screening.

## 2. Experimental Methods

### 2.1. Materials and Instruments

The malachite green standard and potassium ferrocyanide were received from Aladdin (Shanghai, China). Goat anti-mouse IgG was purchased from Sigma-Aldrich (St. Louis, MO, USA). Bovine serum albumin (BSA) was obtained from Amresco (Solon, OH, USA). 3,3′,5,5′-tetramethylbenzidine (TMB) was obtained from Solarbio (Beijing, China). All reagents were of analytical pure grade. Phosphate-buffered saline (PBS, pH 7.4), carbonate coating buffer (CBS, pH 9.6), and washing buffer (PBST, containing 0.05% Tween-20) were prepared using ultrapure water. Scanning electron microscopy (SEM) images were captured using a Tecnai G2 F20 microscope (FEI, Hillsboro, OR, USA). Fourier transform infrared spectrometry (FTIR) was carried out by a Nicolet iS50 spectrometer (Thermo Fisher Scientific, Waltham, MA, USA), and X-ray photoelectron spectroscopy (XPS) was performed with a K-Alpha electron spectrometer (Thermo Fisher Scientific, Waltham, MA, USA). X-ray diffraction (XRD) patterns were recorded on a D8 Advance diffractometer (Bruker, Billerica, MA, USA). Ultraviolet–visible (UV–vis) absorption was assessed by an Evolution 300 spectrophotometer (Thermo Fisher Scientific, Waltham, MA, USA). A microplate reader (BioTek Synergy H1), infrared thermal imaging camera (FLIR E60), and magnetic stirrer (IKA C-MAG HS7) were also employed.

### 2.2. Preparation and Characterization of FGAN@PB NP Nanozyme

**Figure 1:**
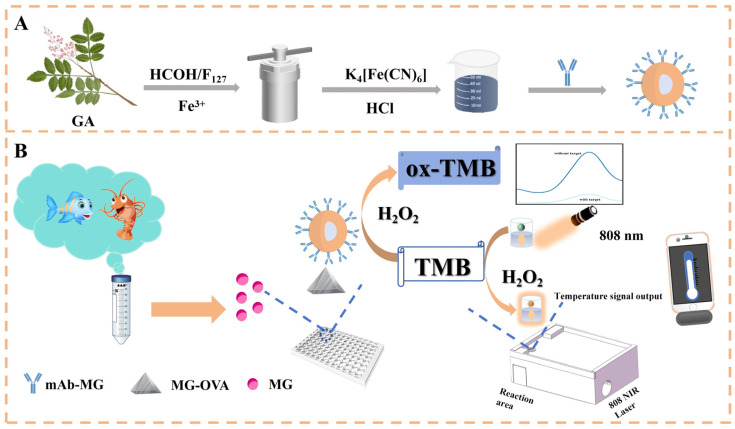
Schematic illustration of the synthesis of FGAN@PB NPs (**A**); principle diagram of the FGAN@PB NP nanozyme-based colorimetric–photothermal dual-mode immunosensor for malachite green detection (**B**).

FGAN nanoparticles (FGAN NPs) were prepared as described by the literature with formaldehyde-assisted a metal–polyphenol ligand crosslinking strategy [26]. As depicted in Figure 1A, Pluronic F127, gallic acid (GA), and ammonia were sequentially dissolved in a mixture of 46 mL water and 8 mL ethanol. Subsequently, 0.38 mL formaldehyde was added, and the solution was stirred for 24 h. Thereafter, Fe(NO_3_)_3_·9H_2_O was introduced, and the reaction was maintained under stirring for an additional 24 h. The resulting mixture was transferred to a reaction vessel and incubated at 100 °C for 24 h. The product was collected by centrifugation and washed five times with ultrapure water. The prepared material was dispersed with ultrapure water, followed by the addition of a 2 mM solution (25 mL) and HCl (0.2 M, 25 mL) under vigorous stirring for 24 h to facilitate the formation of PB nanoparticles on the FGAN NP surfaces. Finally, the product was collected by centrifugation, freeze-dried, and stored as FGAN@PB NP powder. In order to prepare the best performance of FGAN@PB NPs, we optimize the key influencing factors of synthesis. Firstly, the concentration of HCl was fixed at 1 M, and the concentration of K_4_[Fe(CN)_6_] solution was changed from 0 to 6 mM. Alternatively, the concentration of K_4_[Fe(CN)_6_] was fixed at 1 mM, and the concentration of HCl was changed from 0 to 1 M. A continuous-wavelength multifunctional microplate detection platform was employed to record the absorbance at wavelengths of 500–900 nm, with the absorbance value of Prussian blue nanoparticle solution at 710 nm as a reference.

The optical properties, morphology, particle size, surface functional groups, elemental composition, and crystallinity of FGAN@PB NPs were characterized using a combination of a continuous-wavelength multifunctional microplate reader, scanning electron microscopy (SEM), Fourier transform infrared spectrometry (FTIR), X-ray photoelectron spectroscopy (XPS), and X-ray diffraction (XRD).

### 2.3. Catalytic Activity and Steady-State Kinetic Testing of FGAN@PB NP Nanozyme

TMB was used as a chromogenic substrate to evaluate the catalytic activity of FGAN@PB NPs. Activity was confirmed by analyzing the absorption value of 652 nm under various conditions. The effects of pH, temperature, and storage duration on the catalytic stability were assessed using a continuous-wavelength multifunctional microplate reader. For steady-state kinetics, reactions were recorded in the kinetic mode at 652 nm for each substrate concentration. The initial rate (v_0_) was calculated from the linear region of the A_652_–time curve after background correction. Two series were performed: (i) varying TMB (0.1–2.0 mM) at fixed H_2_O_2_ = 1.0 mM, and (ii) varying H_2_O_2_ (0.1–5.0 mM) at fixed TMB = 1.0 mM. The resulting v_0_–[S] data were fitted to the Michaelis–Menten equation to obtain Kₘ and Vₘₐₓ, and they were further checked by Lineweaver–Burk plots.

### 2.4. Photothermal Properties of FGAN@PB NP Nanozyme

The photothermal properties of FGAN@PB NPs and oxidized TMB (ox-TMB) were studied by monitoring the changes in temperature upon 808 nm laser irradiation for 130 s. The optimal irradiation time was established by assessing temperature changes across various concentrations and exposure durations of FGAN@PB NPs. Photothermal stability was further examined over three consecutive “on–off” irradiation cycles.

### 2.5. Preparation of FGAN@PB@Ab1 Probe

The FGAN@PB NPs–anti-MG monoclonal antibody (mAb) conjugate was prepared via a simple mixing method. Specifically, 0.2 mg of FGAN@PB NPs was redispersed in 1 mL of ultrapure water, and 20 μL of anti-MG mAb solution (1 mg/mL) was subsequently added. The mixture was gently agitated for 1 h. Subsequently, 100 μL of 10% BSA was added to block non-specific binding sites. The solution was centrifuged at 7000 r/min for 20 min, the supernatant discarded, and the precipitate dissolved in 1 mL of PBST. The mixture was pipetted evenly and stored at –20 °C for later use. The conjugation of antibodies with signal labels was measured using a NanoDrop 2000c spectrophotometer (Thermo Fisher, Shanghai, China). Ultraviolet–visible (UV–vis) absorption measurements were performed on an Evolution 300 spectrophotometer (Thermo Fisher Scientific, Waltham, MA, USA). ELISA plates were washed using a Multiskan MK2 microplate washer (Thermo Scientific, Waltham, MA, USA). The coupling efficiency = [(total antibodies) − (supernatant antibodies)]/(total antibodies) × 100%.

### 2.6. Procedure of the Colorimetric–Photothermal Immunosensor for Malachite Green Detection

The immunosensor is based on a competitive immunoreaction between free malachite green (MG) in the sample and the MG-OVA coated on the microplate for binding to the FGAN@PB@Ab1 probe. With an increasing MG concentration, fewer nanoprobes bind to the coated antigen, resulting in reduced nanozyme-catalyzed oxidation of TMB in the presence of H_2_O_2_. This leads to a concentration-dependent decrease in both the colorimetric absorbance at 450 nm and the photothermal signal under NIR irradiation, thereby enabling quantitative detection of MG through the construction of calibration curves.

The procedure for detecting MG using the dual-mode immunosensor was as follows: The coated antigen (MG-OVA, 100 μL/well), diluted 2000-fold in carbonate buffer (0.1 M, pH 9.6), was added to a black microplate and incubated for 12 h at 4 °C. The plate was then washed twice with PBST buffer. Subsequently, 120 μL of blocking buffer was added to each well and incubated at 37 °C for 2 h. After discarding the blocking buffer, the plate was stored at 4 °C for future use.

Different concentrations of MG (50 μL, in PBS buffer) and 50 μL of FGAN@PB@Ab1 (5 μg/mL) were sequentially added to the coated microplate. The plate was incubated at 37 °C for 30 min, then washed five times with a plate washer. Next, 100 μL of substrate solution (HAc-NaAc buffer containing 1 M H_2_O_2_ and 0.4 mM TMB) was added to each well and incubated for 20 min at 37 °C.

For photothermal analysis, the prepared microplate strips were placed in a custom fixture designed and manufactured using a 3D printer (Figure 1). The strips were irradiated with an NIR laser (808 nm, 1.5 W/cm^2^) for 150 s. A portable NIR imaging camera was used to monitor the solution’s temperature signal. The standard curve was generated by plotting the temperature signal against the logarithm of MG concentration. The recorded temperature signal was used to calculate the MG concentration from the calibration curve.

The chromogenic reaction was stopped by adding H_2_SO_4_ (10%) for quantification using the colorimetric mode. The absorbance values of the colorimetric solution in each well were immediately measured at 450 nm using a microplate reader. The antibody binding rate was calculated as B/B_0_, where B_0_ is the absorbance value at 450 nm in the absence of MG, and B is the absorbance value at 450 nm in the presence of MG. The standard curve was generated by fitting the logarithmic concentrations of MG with their corresponding B/B_0_ values using a four-parameter logistic regression model. The B/B_0_ values were used to determine the MG concentration from the calibration curve.

### 2.7. Detection of Actual Samples

Five types of aquatic product samples—grass carp, bass, mandarin fish, *Penaeus vannamei*, and aquaculture water—were selected for analysis. Sample pretreatment was conducted according to national standard methods for the determination of MG with some modifications [27]. The detailed sample pretreatment steps were performed as follows: After peeling and shelling, the edible part of pufferfish and snail were cut into small pieces, then crushed. The mixture was homogenized into a paste using a tissue homogenizer. An amount of 5 g of homogenized sample was weighed into a 50 mL centrifuge tube, followed by the addition of 10 mL acetonitrile. The mixture was subjected to ultrasonic extraction for 2 min and homogenized at 8000 r/min for 30 s, then centrifuged at 4000 r/min for 5 min. The supernatant was transferred to a 50 mL beaker and evaporated to near dryness using a sample concentrator. The residue was reconstituted with 25 mL PBS (0.01 M, pH 7.4) to obtain the sample solution, and 50 μL of the pretreated sample solution was used to replace the MG standard, then subjected to the detection procedure described above (Section 2.6).

### 2.8. Validation by LC-MS/MS

In order to verify the accuracy and reliability of the dual-mode immunosensor, the concentration of MG in the spiked sample (spiked levels: 5, 10, and 15 ng/g of MG) was also determined by LC-MS/MS (national standard method of China for determination of MG in aquatic products GB 19857-2005). The specific parameters and calibration curve (Figure S1) are detailed in the Supplementary Materials.

## 3. Results and Analysis

### 3.1. Synthesis and Characterization of FGAN@PB NPs

**Figure 2:**
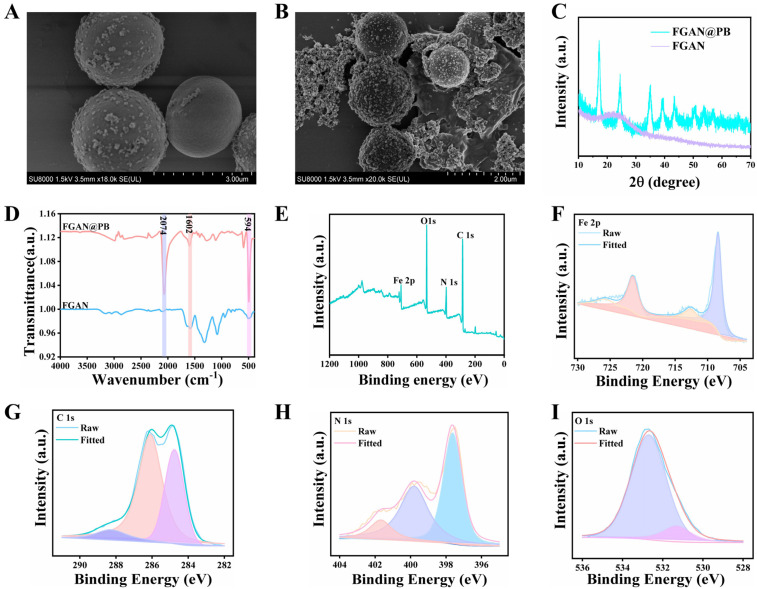
SEM images of FGAN NPs (**A**) and FGAN@PB NPs (**B**); XRD pattern of FGAN and FGAN@PB NPs (**C**); FTIR of FGAN@PB NPs (**D**); XPS survey spectra of FGAN@PB NPs (**E**); XPS spectra of Fe 2p (**F**), C 1s (**G**), N 1s (**H**), and O 1s (**I**) for FGAN@PB NPs.

In this study, FGAN nanospheres were successfully synthesized with a formaldehyde-assisted metal–polyphenol ligand crosslinking method. The morphology of FGAN was examined by SEM, as shown in Figure 2A; FGAN has a uniform spherical morphology with an average size of about 2 μm. Prussian blue nanoparticles (PB NPs) were grown on the FGAN surface by potassium ferricyanide to form FGAN@PB NPs. The pH was adjusted to promote the growth of Prussian blue (PB) nanoparticles on the surface of the FGAN nanospheres. The optimization of key synthesis parameters is shown in Figure S2. Under acidic conditions, K_4_[Fe(CN)_6_] rapidly reacted to form PB nanoparticles on the FGAN surface, resulting in a strong ultraviolet absorption peak at 710 nm, characteristic of PB nanoparticles. When the concentrations of K_4_[Fe(CN)_6_] and HCl were 2 mM (Figures S2A and S2B) and 0.2 M (Figures S2C and S2D), respectively, the absorption peak intensity at 710 nm was both high and stable, indicating that these were the optimal conditions for FGAN@PB NP synthesis. Many characterization methods were applied to further confirm the successful synthesis of the FGAN@PB NPs. SEM was employed to confirm the morphology, as shown in Figure 2B. The FGAN@PB NPs exhibited a spherical morphology with a diameter of around 2 μm, and a series of particles were anchored on the surface of the spherical material, suggesting a composite of PB NPs on the FGAN surface. As shown in Figure 2C, FGAN exhibited an amorphous structure, which did not yield distinct diffraction peaks in the XRD pattern. In contrast, the XRD pattern of FGAN@PB NPs displayed characteristic diffraction peaks at 17.192°, 24.500°, 35.102°, 39.429°, 43.290°, 50.772°, 53.715°, and 57.184°, corresponding to the (200), (220), (400), (420), (422), (440), (600), and (620) crystallographic planes of PB (JCPDS card# 73–0687) [28,29], which indicated the successful formation of crystalline PB nanoparticles on the amorphous FGAN spheres. FTIR spectroscopy (Figure 2D) was employed to investigate the binding states of FGAN and FGAN@PB NPs. For FGAN, a distinct absorption peak at approximately 1602 cm^−1^ was observed, which is attributed to the carboxyl (–COOH) group of gallic acid [30]. After the formation of the composite, the spectrum of FGAN@PB NPs displayed a new characteristic absorption band at 2074 cm^−1^, corresponding to the C≡N stretching vibration in the Fe^2+^–C≡N–Fe^3+^ framework of Prussian blue, and an additional band at 594 cm^−1^, assigned to the bending vibration of this group [31]. Moreover, the carboxyl-related peak exhibited a slight shift and intensity change compared with that of pristine FGAN, suggesting that GA functional groups participated in coordination and facilitated the anchoring of PB nanoparticles on the FGAN spheres. These results collectively confirm the successful integration of FGAN and PB.

To further verify the chemical composition and oxidation states of FGAN@PB NPs, XPS analysis was performed. The full scan spectrum (Figure 2E) indicates the contained C, N, O, and iron elements. Full-region deconvolution (Figure 2F) yields Fe^2+^ 2p_3_/_2_/2p_1_/_2_ at 708.55/721.35 eV and Fe^3+^ 2p_3_/_2_/2p_1_/_2_ at 712.75/724.55 eV [32]. The integrated areas indicate Fe^2+^ = 81.4% and Fe^3+^ = 18.6%. This mixed-valence composition conforms to the Fe^2+^–C≡N–Fe^3+^ framework of Prussian blue and is consistent with the FTIR C≡N band (~2074 cm^−1^) and PB reflections in XRD, confirming the successful formation of FGAN@PB. In the C 1s spectra, the peak at 284.8 eV is assigned to C–C/C=C, whereas the ~286.2 eV component comprises GA-derived C–O/C–N species and overlaps with the expected binding energy of the nitrile carbon (C≡N) in PB; a separate C≡N component is not resolved. No carbonate peak is observed at ~289.0 eV in C 1s, nor a distinct carbonate-type O 1s contribution at ~531.5 eV, offering evidence against significant surface carbonates. The dominant O 1s component at ~532.7 eV is thus primarily attributed to GA-related oxygen [33], with only a minor, if any, contribution from adsorbed H_2_O/–OH. In the N 1s spectra (Figure 2H), the components at ~397.5 eV and ~399.9 eV are characteristic of nitrogen in metal–cyanide ligands and are assigned to coordinated C≡N^−^ in PB with distinct local environments (Fe–N≡C–Fe bridges associated with Fe^2+^/Fe^3+^ neighbors) [34]. A weak high-BE feature at ~401.8 eV is attributed to minor protonated/oxidized nitrogen at surface defect sites. Together with the FTIR C≡N band (~2074 cm^−1^) and the mixed Fe^2+^/Fe^3+^ valence from Fe 2p, these N 1s features corroborate the PB-type Fe–N≡C–Fe coordination in FGAN@PB.

### 3.2. Enzyme-Mimicking Catalytic Activity and Steady-State Kinetic Analysis of FGAN@PB NPs

**Figure 3:**
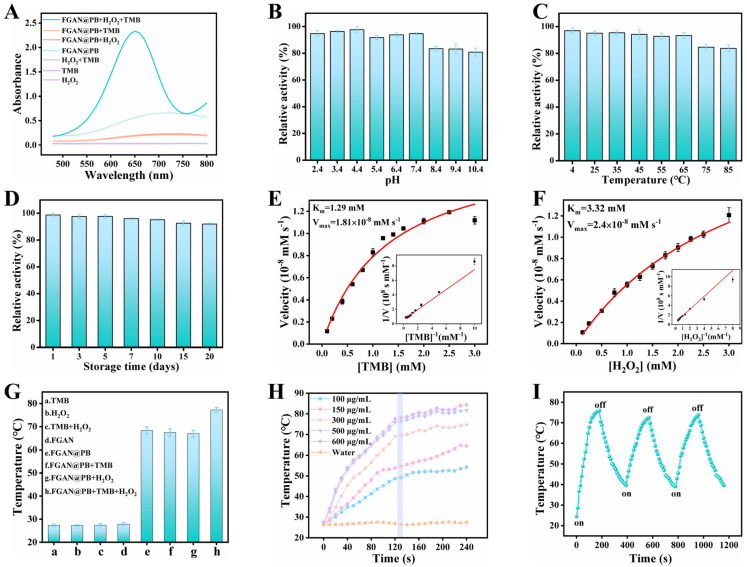
UV-Vis spectra of FGAN NPs catalyzing the oxidation of TMB (**A**); influence of pH (**B**) and temperature (**C**) changes on the catalytic activity of FGAN@PB NPs; storage stability of FGAN@PB NPs (**D**); steady-state kinetic analysis of FTAN@PB as peroxidase mimetic: curve of velocity against the TMB concentration in condition of 1.0 mM H_2_O_2_ (**E**); curve of velocity against the H_2_O_2_ concentration in condition of 1.0 mM TMB (**F**); photothermal effects of different components (**G**); heating curves of FGAN@PB NPs at varying concentrations (100, 150, 300, 500, and 600 μg·mL^−1^), control group: water (**H**); photothermal stability of FGAN@PB NPs over three cycles of laser on/off switching (**I**).

The peroxidase-like catalytic activity of FGAN@PB NPs was confirmed using the chromogenic substrate TMB. In the presence of H_2_O_2_, FGAN@PB NPs catalyzed the oxidation of TMB to produce a blue product with a strong absorption peak at 652 nm (Figure 3A). In contrast, the control group showed no significant absorption at 652 nm, confirming the nanozyme’s peroxidase-mimicking activity. In line with canonical PB nanozymes, the catalytic activity is ascribed to PB domains undergoing Fe(II)/Fe(III) redox cycling in the presence of H_2_O_2_. Fe(II) sites activate H_2_O_2_ in a Fenton-like manner to generate reactive oxidants that convert TMB to ox-TMB; Fe(III) is then reduced back to Fe(II) by the substrate, completing the cycle.

FGAN@PB NPs exhibited remarkable catalytic activity over a wide pH range (Figure 3B) and retained over 83% activity after exposure to various temperatures for 10 min (Figure 3C). Furthermore, the nanozyme preserved its catalytic function even after being stored at room temperature for 20 days (Figure 3D), demonstrating excellent stability and peroxidase-like activity.

The kinetic parameters Km and Vmax of FGAN@PB NPs were obtained by fitting the data to the Michaelis–Menten model (Figure 3E) and confirmed by Lineweaver–Burk double reciprocal plots (Figure 3F). The Km values for TMB and H_2_O_2_ were 1.29 mM and 3.32 mM, respectively, with corresponding Vmax values of 1.81 × 10^−8^ mM·s^−1^ and 2.4 × 10^−8^ mM·s^−1^. To benchmark catalytic affinity, we compared the apparent Michaelis constants (Kₘ) toward TMB and H_2_O_2_ (Table S1). Relative to HRP (literature values: Kₘ(TMB) = 0.434 mM, Kₘ(H_2_O_2_) = 3.70 mM), FGAN@PB displays a higher Kₘ for TMB (lower apparent affinity), but a comparable—slightly lower—Kₘ for H_2_O_2_. Compared with representative peroxidase-like nanozymes, FGAN@PB offers a balanced substrate affinity: its Kₘ(TMB) is lower than Fe–N–C (3.6 mM) and Hemin–Au@MOF (2.67 mM), and it is comparable to Cu NCs (0.648 mM) while higher than Fe_3_O_4_ (0.098 mM), Zn–N–C (0.224 mM), FGN (0.154 mM), and BP/Au (0.417 mM). For H_2_O_2_, FGAN@PB ranks among the best, with Kₘ(H_2_O_2_) = 3.32 mM, which is much lower than Fe_3_O_4_ (154 mM), Fe–N–C (12.2 mM), Cu NCs (29.16 mM), Zn–N–C (40.16 mM), FGN (7.51 mM), and BP/Au (20.69 mM), and which is close to HRP (3.70 mM); only Hemin–Au@MOF (2.58 mM) is slightly lower. Together with its broad pH/temperature tolerance and the additional photothermal readout not available to HRP, these data indicate that FGAN@PB NPs combine robust operability with competitive substrate affinity, especially for H_2_O_2_.

### 3.3. Investigation of the Photothermal Properties of FGAN@PB NPs

The photothermal effect of the FGAN@PB NPs was evaluated by monitoring temperature changes under 808 nm laser irradiation (Figure 3G). A significant temperature increase was observed in the presence of FGAN@PB NPs. When FGAN@PB NPs, H_2_O_2_, and TMB were simultaneously present, the system exhibited the highest temperature rise. In contrast, the control group showed negligible changes, confirming the nanozyme’s effective photothermal conversion capability.

In experiments with varying FGAN@PB NP concentrations (100, 150, 300, 500, and 600 μg/mL), the temperature increased progressively with prolonged irradiation and stabilized after 130 s (Figure 3H). Higher nanoparticle concentrations produced greater temperature elevations, demonstrating a positive correlation between photothermal efficiency and nanozyme concentration. Moreover, photothermal stability was assessed by three laser irradiation cycles (Figure 3I). The magnitude of the temperature increases in each cycle indicates the excellent photothermal stability of FGAN@PB NPs, reinforcing their applicability as a novel photothermal agent.

### 3.4. Evaluation of the Conjugation of FGAN@PB NPs with Antibodies

**Figure 4:**
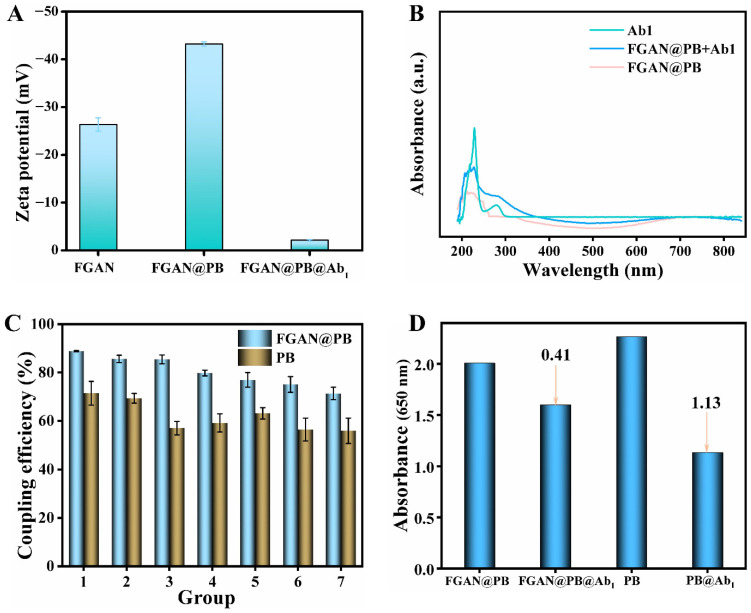
Zeta potentials of FGAN, FGAN@PB, and FGAN@PB@Ab1 (**A**); UV-Vis spectra of FGAN@PB, Ab1, and FGAN@PB@Ab1 (**B**); coupling efficiency of PB and FGAN@PB NPs with anti-MG mAbs (Groups 1–6: antibody concentrations of 0.5, 1, 2, 3, 4, 5, and 6 μg·mL^−1^) (**C**); catalytic activity of PB and FGAN@PB NPs before and after conjugation with anti-MG mAbs (**D**).

A simple mixing method was employed to conjugate monoclonal anti-antibodies (anti-MG mAbs) to the surface of FGAN@PB NPs, thereby forming the nanoprobe FGAN@PB@Ab1, which possessed specific monoclonal antibody recognition capability. The zeta potential and UV-Vis absorption spectra of FGAN, FGAN@PB NPs, and FGAN@PB@Ab1 were analyzed, as presented in Figure 4. Following PB complexation, the zeta potential of FGAN@PB NPs decreased relative to that of FGAN. Prior to antibody labeling, the zeta potential of FGAN@PB NPs was −43.2 mV; after labeling, the zeta potential of the nanoprobe increased to −2.14 mV. This shift indicates electrostatic attraction between the negatively charged FGAN@PB NPs and the positively charged antibodies (Figure 4A). In the UV-Vis spectrum (Figure 4B), FGAN@PB NPs showed no distinct absorption peaks, whereas Ab1 exhibited a characteristic absorption peak at 280 nm. The presence of this peak in FGAN@PB@Ab1 further confirmed the successful conjugation of the nanozyme with the antibody.

Compared with conventional Prussian blue nanoparticles (PB NPs), FGAN@PB NPs demonstrated a superior antibody conjugation efficiency. As shown in Figure 4C, the conjugation efficiency of PB NPs ranged from 55% to 71%, whereas FGAN@PB NPs achieved a conjugation efficiency of up to 88.9%. This enhancement is likely due to the excellent biocompatibility conferred by gallic acid and the electrostatic adsorption properties of FGAN@PB NPs. Zeta potential measurements further corroborated the contribution of electrostatic interactions to the conjugation process. Furthermore, after conjugation with anti-MG mAbs, the catalytic activity of PB NPs toward TMB was significantly diminished (Figure 4D), suggesting that the coupling of antibody protein and BSA blocking interfered with the active catalytic sites of the nanozyme. In contrast, the catalytic activity of FGAN@PB NPs was relatively less sensitive to these biomolecular interactions.

### 3.5. Optimization of Key Parameters for the Immunosensor

To optimize the performance of the immunosensor, key experimental parameters such as the reconstitution buffer type for FGAN@PB@Ab1, pH, the amount of anti-MG mAbs used for labeling, the concentration of the coated antigen, and the incubation time were systematically investigated. As shown in Figure S3A, various buffer solutions were tested for their effects on the OD450 values. The results indicated that Hepes buffer provided the highest OD450 signal and was thus the most suitable for reconstituting FGAN@PB@Ab1. The impact of pH was also evaluated (Figure S3B), revealing that extremely acidic or alkaline conditions significantly influenced the nanoprobe, whereas weakly acidic or neutral conditions had minimal effects. The highest OD450 value was recorded at pH 6.4.

The optimal amount of anti-MG mAbs for labeling and the appropriate dilution of the coated antigen were subsequently determined. As shown in Figure S3C,D, the optimal antibody concentration for labeling was 5 μg/mL, and the optimal coated antigen dilution ratio was 1:2000. Furthermore, enzymatic reaction time was recognized as a critical factor influencing system performance. An insufficient incubation may lead to incomplete oxidation of the catalytic substrate, while an excessive time may compromise efficiency and delay detection. In this study, the effects of incubation times of 10, 20, 30, 40, 50, and 60 min on the OD450 signal were assessed (Figure S3E). The OD450 signal increased from 10 to 30 min and then declined gradually, indicating that 30 min was the optimal incubation time.

### 3.6. Performance of the Dual-Mode Immunosensor

**Figure 5:**
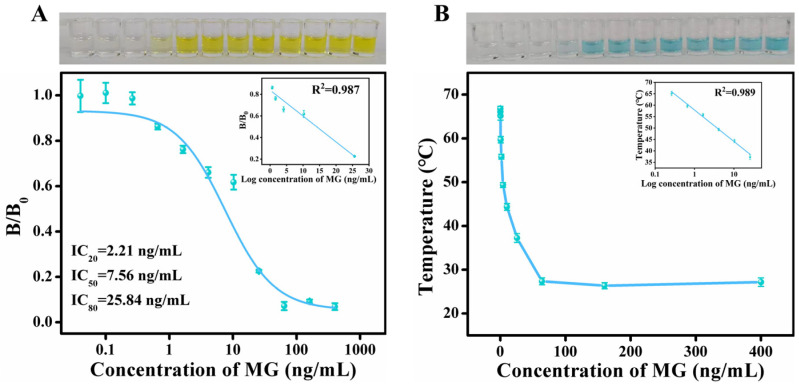
Standard curve of colorimetric immunoassay for MG detection, inset: linear range, photo corresponding to colorimetric immunoassay (top) (**A**); standard curve of photothermal immunoassay, inset: linear range, photo before photothermal immunoassay (top) (**B**).

Under optimized conditions, standard calibration curves of both the colorimetric and photothermal immunosensor modes for malachite green detection were established. As shown in Figure 5A, a standard curve was plotted using a four-parameter nonlinear equation, with MG concentration on the x-axis and B/B0 on the y-axis. The assay exhibited an IC_50_ value of 7.56 ng/mL within a concentration range of 2.21–25.84 ng/mL; moreover, there was a good linear relationship between the B/B0 value and the logarithmic concentration of malachite green.

A standard curve for the photothermal signal was also established. In the concentration range of 0.262–25.6 ng/mL, both color intensity and solution temperature decreased with an increasing MG concentration. The linear regression between temperature (y) and the logarithm of MG concentration (x) was expressed as y = 57.87 − 13.75x, with a calculated limit of detection (LOD) of 0.31 ng/mL based on the 3σ/m criterion (Figure 5B), where σ is the blank measurements’ standard deviation and m represents the slope of the calibration curve.

Compared with conventional ELISA, the present approach simplifies the detection procedure by directly conjugating the antibody to the nanozyme, thereby shortening the reaction time and enabling rapid detection. Moreover, the photothermal immunoassay results were consistent with those of the colorimetric assay, which can achieve mutual confirmation. The portability of the photothermal detection method also offers promising potential for on-site analysis of malachite green residues.

### 3.7. Detection of MG in Real Samples

To evaluate the practical applicability of the developed colorimetric–photothermal dual-mode immunosensor, recovery tests were conducted using malachite green-spiked real food samples. The results were validated against those obtained using LC-MS/MS, as presented in Table 1. For the colorimetric method, recovery rates are between 86.4% and 102.6%, with coefficients of variation (CV) below 12.5%. In the photothermal mode, recovery rates are between 82.9% and 99.9%, with CVs below 12.6%.

Additionally, the detection results obtained from both the colorimetric and photothermal methods were consistent with those of LC-MS/MS, demonstrating that the immunosensor provided accurate and reliable results. These findings confirm the potential of the proposed dual-mode immunosensor for practical applications in food safety monitoring.

## 4. Conclusions

In this study, a dual-mode immunosensor based on FGAN@PB NP nanozymes was developed, demonstrating high sensitivity and a dual-signal output for the detection of malachite green. The FGAN@PB NP nanozymes exhibited excellent enzyme-like activity and photothermal conversion efficiency, attributed to the synergistic effects of the metal–phenolic network and Prussian blue, effectively overcoming the stability limitations of conventional horseradish peroxidase (HRP). In the colorimetric mode, the sensor achieved an IC50 value of 7.56 ng/mL with a linear detection range of 2.21–25.84 ng/mL. In the photothermal mode, the linear detection range was 0.262–25.6 ng/mL, with an LOD of 0.31 ng/mL (y = 57.87 − 13.75x). The integration and mutual validation of both detection modes significantly enhanced the reliability of the results. Validation using LC-MS/MS confirmed the practical applicability of the sensor, offering a promising tool for the rapid on-site screening of malachite green in aquaculture samples.

## Figures and Tables

**Table 1:** Recoveries of MG from spiked water and food samples base on colorimetric–photothermal dual-mode immunosensor and LC-MS/MS.

Sample		Colorimetric Mode			Photothermal Mode			LC-MS/MS	
	Spiked Level (ng/mL or g)	Measured (ng/mL or g) (*X* ±SD) ^a^	Recovery (%)	CVs (%)	Measured (ng/mL or g) (*X* ± SD) ^a^	Recovery (%)	CVs(%)	Measured (ng/mL or g) (*X* ± SD) ^a^	Recovery (%)
aquaculture water	5	5.1 ± 0.3	102.1	4.9	4.7 ± 0.3	94.6	5.0	5.3 ± 0.1	105.7
	10	9.4 ± 0.4	94.2	3.8	9.5 ± 0.7	95.1	7.5	10.4 ± 0.2	103.5
	15	14.1 ± 0.2	94.1	1.2	14.4 ± 1.0	95.8	6.7	16.0 ± 0.3	106.6
mandarin fish	5	4.7 ± 0.2	93.2	3.3	4.7 ± 0.2	93.0	5.0	4.9 ± 0.2	98.4
	10	10.3 ± 0.6	102.6	5.7	10.0 ± 0.8	99.5	7.6	9.5 ± 0.2	94.7
	15	14.7 ± 0.7	97.9	4.8	14.8 ± 0.7	98.4	4.5	14.7 ± 0.3	98.2
bass	5	5.0 ± 0.5	100.6	10.4	4.5 ± 0.6	89.9	12.6	4.9 ± 0.2	98.0
	10	9.1 ± 0.4	91.4	3.7	10.0 ± 0.6	99.9	5.9	9.5 ± 0.2	95.2
	15	14.7 ± 0.6	97.7	4.16	12.5 ± 0.6	83.3	4.5	15.1 ± 0.7	100.5
grass carp	5	4.3 ± 0.2	86.4	5.1	4.1 ± 0.4	82.9	8.7	4.8 ± 0.1	95.5
	10	9.3 ± 0.5	93.1	5.3	8.7 ± 0.9	87.1	10.4	9.5 ± 0.6	94.9
	15	14.0 ± 0.8	93.1	5.7	14.6 ± 1.0	97.4	6.7	13.7 ± 0.3	91.6
*Penaeus vannamei*	5	4.3 ± 0.5	86.5	10.4	4.2 ± 0.5	84.5	12.5	4.7 ± 0.1	94.2
	10	9.9 ± 1.2	98.6	12.5	9.8 ± 0.7	97.8	7.6	10.2 ± 0.2	101.8
	15	13.3 ± 0.9	88.3	7.1	14.2 ± 0.6	94.7	4.2	14.9 ± 0.3	99.4

^a^ (*X* represents the average value; SD stands for standard deviation).

## Data Availability

The datasets will be made available upon request.

## Supplementary Materials

The Supplementary Materials for this article can be found online at https://www.mdpi.com/article/10.3390/bios15110719/s1. Figure S1. Calibration curve for MG detection by LC-MS/MS. Figure S2. Optimization of synthesis conditions of FGAN@PB NPs: optimization of potassium ferrocyanide concentration (A,B) and hydrochloric acid concentration (C,D). Figure S3. Optimization of immunosensor working parameters: resuspension buffer for the FGAN@PB@Ab1 probe (A) and buffe pH (B); anti-MG mAb concentration for labeling (C); coated antigen concentration (D); enzymatic reaction time (E). Table S1. Comparison of the Km of FGAN@PB NPs with those of HRP and representative peroxidase-like nanozymes, references [35,36,37,38,39,40].
