# Supplementary material for: FGAN@PB NP Nanozyme-Based Colorimetric–Photothermal Dual-Mode Immunosensor for Malachite Green Detection
Source: Biosensors (Basel). 2025 Oct 30;15(11):719. doi: 10.3390/bios15110719 (PMC12649948; doi:10.3390/bios15110719)
Supplement: Supplementary file 1 [file biosensors-15-00719-s001.zip › biosensors-3873305-supplementary.pdf]

biosensors

MDPI

Supporting Information

FGAN@PB NP Nanozyme-Based Colorimetric–Photothermal Dual-Mode Immunosensor for Malachite Green Detection

Min-Fu Wu [1,2], Jing-Min Li [2,†], Sha Li [1,3,*], Min-Hua Wu [4], Ri-Sheng Chen [2], Yan-Can Liu [1], Jian-Nan Liu [1], Zhen-Lin Xu [2], Yi-Chao Yang [5], Jia-Dong Li [1], Qing-Yi Lei [1], Si-Min Zhan [1] and Lin Luo [2,*]

1 Department of Food Science, Foshan Polytechnic, Foshan 528137, China; wmf@fspt.edu.cn (M.-F.W.); liuyancan@fspt.edu.cn (Y.-C.L.); liujiannan@fspt.edu.cn (J.-N.L.); ljdong135@fspt.edu.cn (J.-D.L.); lqy2025@fspt.edu.cn (Q.-Y.L.); zsm2025@fspt.edu.cn (S.-M.Z.)
2 Guangdong Provincial Key Laboratory of Food Quality and Safety, College of Food Science, South China Agricultural University, Guangzhou 510642, China; lijm@bioeasy.com (J.-m.L.); 20241145003@stu.scau.edu.cn (R.-S.C.); xzlin@scau.edu.cn (Z.-L.X.)
3 School of Health Sciences Research, Research Institute for Health Sciences, Chiang Mai University, Chiang Mai 50200, Thailand
4 Department of Histology and Embryology, Guangdong Medical University, Zhanjiang 524023, China; wugdmczp@gdmu.edu.cn
5 School of Public Health, Guangzhou Medical University, Guangzhou, Guangdong 511436, China; yichaoyang@gzhmu.edu.cn
* Correspondence: lisha199007@163.com (S.L.); lin.luo@scau.edu.cn (L.L.)
† Co-first author: lijingmin1109@163.com.

Liquid chromatography–tandem mass spectrometry/mass spectrometry (LC-MS/MS) for MG detection

The dual-mode immunosensor was validated by LC-MS/MS, as described in the national standard method of China for the determination of MG in aquatic products GB GB/19857-2005. Briefly, 2 μL of the prepared sample was injected into a QTRAP™ 4500 system equipped with a C18 chromatographic column (100 mm×2.1mm, 1.7 μm particle size, column temperature 40 °C) and separated at a flow rate of 300 μL/min (mobile phase A, 0.2% acetic acid; mobile phase B, acetonitrile). The MS parameters were as follows: desolation temperature at 550 °C; desolvation gas (N_2), flow rate at 10 L/min; ion spray voltage at 5500 V. The analytes were identified by parent/daughter ions as well as peak retention times in comparison to the standards. The $[M + H]^+$ at m/z 329 was the parent ion of MG. The daughter ion at m/z 313 was used for quantitation. Figure S1 shows the calibration curve for benzocaine quantification by LC-MS/MS.

Regression Equation: y = 0.92504 x + -0.00587 (r = 0.99857) (weighting: 1 / x)

Expected Concentration	Number of Values	Mean Calculated Concentration	% Accuracy	Std. Deviation	%CV
0.200	1 of 1	0.22	112.5	N/A	N/A
1.000	1 of 1	1.01	101.0	N/A	N/A
2.000	1 of 1	1.93	96.4	N/A	N/A
5.000	1 of 1	4.86	97.1	N/A	N/A
10.000	1 of 1	9.67	96.7	N/A	N/A
20.000	1 of 1	18.42	92.1	N/A	N/A
50.000	1 of 1	52.09	104.2	N/A	N/A

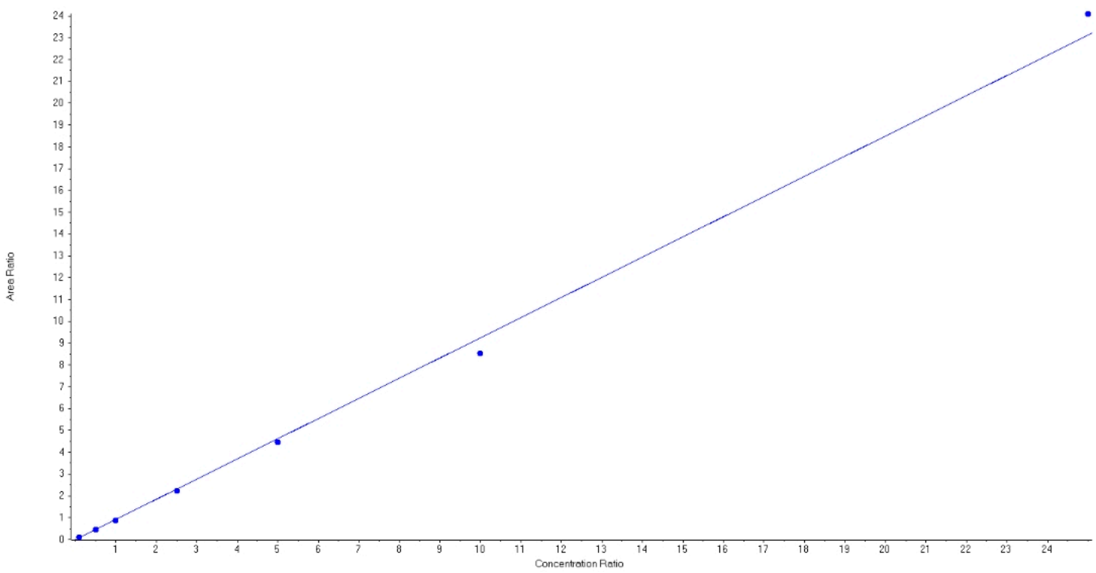

Figure S1. Calibration curve for MG detection by LC-MS/MS.

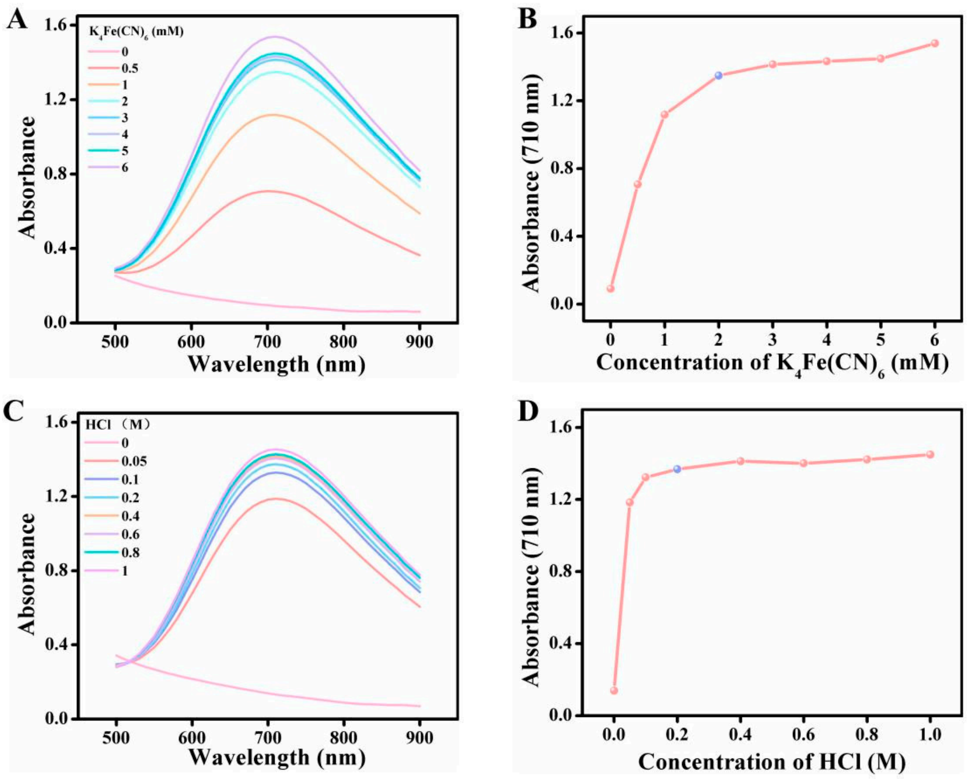

Figure S2. Optimization of synthesis conditions of FGAN@PB NPs: optimization of potassium ferrocyanide concentration (A, B) and hydrochloric acid concentration (C, D).

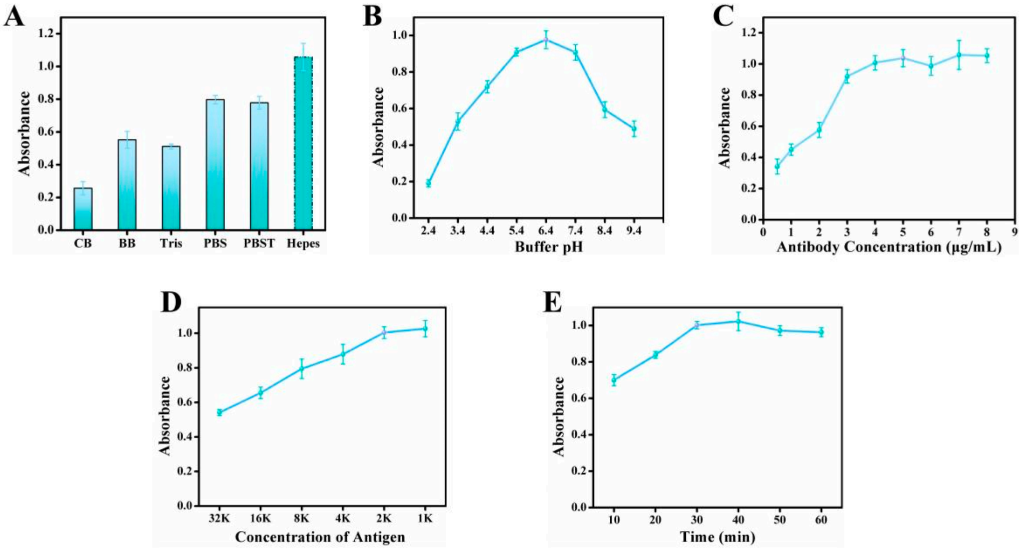

Figure S3. Optimization of immunosensor working parameters: resuspension buffer for the FGAN@PB@Ab1 probe (A) and buffer pH (B); anti-MG mAb concentration for labeling (C); coated antigen concentration (D); enzymatic reaction time (E).

Table S1. Comparison of the Km of FGAN@PB NPs with those of HRP and representative peroxidase-like nanozymes.

Catalyst	Substrate	Km	Reference
HRP	TMB	0.434	[36]
	H_2O_2	3.70	
Fe_3O_4 NPs	TMB	0.098	[36]
	H_2O_2	154	
Fe-N-C	TMB	3.6	[38]
	H_2O_2	12.2	
Cu NCs	TMB	0.648	[37]
	H_2O_2	29.16	
Hemin-Au@MOF	TMB	2.67	[40]
	H_2O_2	2.58	
Zn-N-C	TMB	0.224	[39]
	H_2O_2	40.16	
FGN	TMB	0.154	[26]
	H_2O_2	7.51	
BP/Au	TMB	0.417	[35]
	H_2O_2	20.69	
FGAN@PB NPs	TMB	1.29	This work
	H_2O_2	3.32	

References

1. Ding, L., Shao, X., Wang, M., Zhang, H., & Lu, L. (2021). Dual-mode immunoassay for diethylstilbestrol based on peroxidase activity and photothermal effect of black phosphorus-gold nanoparticle nanohybrids. *Analytica Chimica Acta, 1187*.
2. Gao, L., Zhuang, J., Nie, L., Zhang, J., Zhang, Y., Gu, N., Wang, T., Feng, J., Yang, D., Perrett, S., & Yan, X. (2007). Intrinsic peroxidase-like activity of ferromagnetic nanoparticles. *Nature Nanotechnology, 2*(9), 577-583.
3. Hu, L., Yuan, Y., Zhang, L., Zhao, J., Majeed, S., & Xu, G. (2013). Copper nanoclusters as peroxidase mimetics and their applications to H2O2 and glucose detection. *Analytica Chimica Acta, 762*, 83-86.
4. Jiao, L., Xu, W., Yan, H., Wu, Y., Liu, C., Du, D., Lin, Y., & Zhu, C. (2019). Fe–N–C Single-Atom Nanozymes for the Intracellular Hydrogen Peroxide Detection. *Analytical Chemistry, 91*(18), 11994-11999.

5. Li, Y., Liu, S., Yin, X., Wang, S., Tian, Y., Shu, R., Jia, C., Chen, Y., Sun, J., Zhang, D., Zhu, M., & Wang, J. (2022). Nature-inspired nanozymes as signal markers for in-situ signal amplification strategy: A portable dual-colorimetric immunochromatographic analysis based on smartphone. *Biosensors and Bioelectronics, 210*.
6. Xu, B., Wang, H., Wang, W., Gao, L., Li, S., Pan, X., Wang, H., Yang, H., Meng, X., Wu, Q., Zheng, L., Chen, S., Shi, X., Fan, K., Yan, X., & Liu, H. (2019). A Single-Atom Nanozyme for Wound Disinfection Applications. *Angewandte Chemie International Edition, 58*(15), 4911-4916.
7. Zhang, L., Fan, C., Liu, M., Liu, F., Bian, S., Du, S., Zhu, S., & Wang, H. (2018). Biominerized gold-Hemin@MOF composites with peroxidase-like and gold catalysis activities: A high-throughput colorimetric immunoassay for alpha-fetoprotein in blood by ELISA and gold-catalytic silver staining. *Sensors and Actuators B: Chemical, 266*, 543-552.
